# Letter to the Editor Concerning the Article: Impact of Quinolone Prophylaxis on Spontaneous Bacterial Peritonitis and Mortality in Cirrhosis Patients: A Systematic Review and Meta‐Analysis of Randomized Controlled Trials

**DOI:** 10.1002/jgh3.70451

**Published:** 2026-07-25

**Authors:** Alejandro Chen Liang, Yeison Cruz Castillo, Lorena M. Murrieta, Jesus Eleazar Angulo Durazo, Hector Jonan Flores Uribe

**Affiliations:** ^1^ Centro de Estudios Universitarios Xochicalco Campus Tijuana Tijuana México; ^2^ Universidad Autónoma de Santo Domingo Santo Domingo República Dominicana; ^3^ Universidad del Valle de México Hermosillo México

To the Editor,

1

We read with great interest the recent meta‐analysis evaluating quinolone prophylaxis for the prevention of spontaneous bacterial peritonitis (SBP) and mortality in patients with cirrhosis by Malvi et al. [1]. We commend the authors for addressing a clinically important question and for conducting a systematic review and meta‐analysis of randomized controlled trials in this field.

First, regarding the assessment of risk of bias, the authors reported the use of the Cochrane Risk of Bias 2.0 (RoB 2) tool. However, based on the domain‐level assessments presented, several studies classified as overall “low risk” include at least one domain rated as “some concerns,” particularly in relation to missing outcome data. According to RoB 2 guidance, an overall “low risk” judgment requires all domains to be rated as low risk; the presence of any domain with “some concerns” would preclude this classification. Clarification on how overall judgments were derived would help contextualize these study‐level assessments [2, 3, 4].

Second, while the review focuses on randomized controlled trials, clarification regarding the eligibility criteria for comparator groups would be valuable. Although quinolone prophylaxis is defined as the intervention of interest, the comparator is not explicitly specified. As included studies are predominantly presented as placebo‐controlled, it would be helpful to clarify whether trials with active comparators were considered during study selection, as this may influence the scope and interpretability of the pooled estimates.

Additionally, recurring considerations within the same risk‐of‐bias domain across multiple studies may have implications for the overall certainty of the evidence and its interpretation.

Overall, further clarity in methodological reporting, particularly regarding risk of bias assessment and study selection decisions, may help strengthen the transparency and interpretability of the findings. In line with PRISMA 2020 Statement recommendations, transparent reporting of these processes is essential to ensure reproducibility and allow readers to fully assess the validity of the findings [2].

## Conflicts of Interest

The authors declare no conflicts of interest.

## Data Availability

Data sharing not applicable to this article as no datasets were generated or analysed during the current study.
